# An ultrasound-guided minimally invasive treatment for vaginal vault collections following major gynaecological surgery: a case series

**DOI:** 10.3389/fsurg.2026.1661216

**Published:** 2026-05-11

**Authors:** Nicholas Anson, Lorraine S. Kasaven, Shaun Haran, Anita Mitra, David Constable-Phelps, Maya Al-Memar, Benjamin P. Jones, Srdjan Saso, Joseph Yazbek

**Affiliations:** 1 Division of Surgery and Cancer, Institute of Reproductive & Developmental Biology, Imperial College London, Hammersmith Hospital Campus, London, United Kingdom; 2 University Hospital Southampton NHS Foundation Trust, Southampton, United Kingdom; 3 The Lister Fertility Clinic, The Lister Hospital, London, United Kingdom

**Keywords:** drainage, hysterectomy, minimally invasive, ultrasound-guidance, vaginal vault collections

## Abstract

**Introduction:**

Post-hysterectomy vaginal vault collections, often infected haematomas, cause significant morbidity requiring resource-intensive and invasive management. Our proposed method for vault drainage, a novel ultrasound-guided technique, aims to provide a minimally invasive treatment for refractory collections. This is a proof-of-concept case series that describes the technique and evaluates its potential efficacy.

**Methods:**

Retrospective case series of nine patients with vaginal vault collections (eight infected haematomas, one lymphocyst) refractory to, or unsuitable for conservative management with intravenous antibiotics. Transvaginal drainage with blunt sponge holding forceps was performed under transabdominal ultrasound-guidance, taking 3–5 min with minimal discomfort. Prior transvaginal ultrasound was performed to confirm collection anatomy. The primary outcomes were symptom resolution and inflammatory marker reduction. All patients were seen and treated at Hammersmith Hospital, Imperial College Healthcare NHS Trust, London, United Kingdom.

**Results:**

All patients had their vaginal vault collection successfully drained with subsequent resolution of their symptomatic illness (e.g., pyrexia, pain, discharge). One patient required repeat drainage. Inflammatory markers decreased significantly post-drainage. No procedure-related complications occurred.

**Discussion:**

The proposed procedure treated all patients' vaginal vault collections. This technique offers a minimally invasive, ultrasound-guided alternative to interventional radiology or surgery. This may particularly benefit high-risk patients such as we describe (e.g., high BMI, complex surgery, cancer diagnoses) who are less suitable for invasive intervention.

**Conclusions:**

We present an ultrasound-guided, minimally invasive technique for the treatment of refractory post-hysterectomy vaginal vault collections that avoids the need for sharp instruments or surgical intervention.

## 1 Introduction

Following hysterectomy, gravity-dependent pelvic collections can accumulate at the vaginal vault. Pelvic collections, especially haematomas, are a recognised complication following all hysterectomy approaches with rates of vault haematomas noted to have a variable incidence in the literature. Earlier studies noted this complication more frequently, with rates as high as 98% on routine scanning post vaginal hysterectomy (1–5).

Pelvic haematomas remain more commonly associated with vaginal hysterectomy, followed by minimally invasive and abdominal approaches (6, 7). Susceptibility of these haematomas to infection can lead to significant morbidity for the patient. While rates of sonographically diagnosed haematoma are high, febrile morbidity is less common. For example, after vaginal hysterectomy, 20%–25% of patients may have an incidental vault haematoma when routinely scanned, with only 7%–9% developing symptoms such as fever or pain requiring intervention (8, 9).

When considering all routes of hysterectomy, use of modern surgical techniques have combined rates of infected pelvic haematoma as low as 2.2% (7). While a recent Cochrane review on surgical approaches to benign hysterectomy did not find a difference in the rates of pelvic haematoma between surgical routes, the topic of infected vault haematoma was not specifically addressed. However, the review did suggest lower overall rates of infection with vaginal and laparoscopic hysterectomy, when compared to abdominal hysterectomy (10).

More broadly, surgical site infections (SSIs), including organ space infections, remain a major cause of post-operative complications and are estimated to account for 20% of all hospital acquired infections (11, 12). These infections may lead to post-operative sepsis which remains a major cause of morbidity and mortality, and is estimated to occur in 1%–3% of patients in the developed world and significantly more in the developing world (12–15). There are established risk factors when considering all types of SSI following major gynaecological surgery which include obesity, diabetes, surgery for cancer, complex oncological surgery, intra-operative blood transfusion, prolonged surgical time and surgical route (10, 12, 16–18). Timely recognition and treatment of gynaecological SSIs is essential as it is likely the manner in which these are addressed, as opposed to the complication rate alone, that affects mortality (19).

Most vaginal vault haematomas can be managed expectantly, or with antimicrobials and interval ultrasonography if pain or febrile morbidity develop. Patients with persistently raised inflammatory markers or abnormal clinical observations despite adequate intravenous broad-spectrum antimicrobials will require drainage. Described methods include computed tomography (CT) guided percutaneous drainage, ultrasound-guided drainage (transvaginal, transrectal and transperineal), and the manual opening of vaginal vault sutures (20). Where these options are not feasible or available, a return to theatre may be warranted.

Ultrasound-guided procedures are increasingly common in many surgical fields, including gynaecology (21–23). While gynaecological ultrasound is routine practice for investigating benign and malignant pelvic conditions, intra-operative use is a more recent development (21, 23). Transabdominal and transvaginal ultrasound-guidance are described for multiple use cases including removal of irretrievable intrauterine devices, surgical management of miscarriage and termination, embryo transfer, operative hysteroscopy, laparoscopic fibroid identification, intracavitary brachytherapy, fertility sparing ovarian cystectomy and ovarian wedge resection (24–31). Potential indications and uptake will likely continue to grow as awareness and training improve alongside more advanced laparoscopic and robotic equipment for ultrasound-guidance becoming commonplace (21–23).

Given that patients benefit from avoiding invasive management of vault collections, we describe in this proof-of-concept case series an ultrasound-guided technique that may provide clinicians with a simple and well tolerated method for drainage. Previous studies provide limited detail on the expected patient response and recovery following other techniques; therefore, this case series may offer a useful benchmark for future comparative studies.

## 2 Materials and methods

Here we present a case series describing an effective method performed locally for the treatment of nine patients with complex post-operative pelvic collections that include: infected vaginal vault haematomas (*n* = 8) and pelvic lymphocyst (*n* = 1). The primary outcomes were the resolution of symptomatic illness and reduction in inflammatory markers.

All patients were operated on at a supra-regional tertiary gynae-oncology hospital due to their significant medical and surgical complexity, which may have predisposed them to the formation of symptomatic vault collections (Table 1). Six patients were American Society of Anesthesiologists (ASA) grade 3 and three patients were ASA grade 2. The ASA grade 2 patients had factors contributing to their complexity including a large mass posing surgical challenges (*n* = 1), previous gynaecological cancer surgery (*n* = 1), and advanced endometriosis (*n* = 1).

**Table 1:** Patient characteristics.

Patient	Age	BMI	ASA	Procedure	Indication	Co-morbidities	Intraoperative Comments or Complications
*1*	26	35	3	TLH BSO and open repair of rectal injury	PMDD	Recurrent VTE with lifelong	Pouch of Douglas endometriotic nodules
					Endometrial hyperplasia	edoxaban	Rectal injury requiring lower midline laparotomy for repair
						Spondyloarthritis	
						Vasculitis	
						Splenectomy	
*2*	75	22	2	TLH BSO	Large left adnexal mass (benign histology)	1st degree heart block	Significant adhesions with large bowel adherent to uterine fundus
						Hypertension	Jejunal perforation with primary trocar insertion, repaired per vagina
							Adnexal mass within the broad ligament attached to pelvic sidewall
*3*	47	20	3	TAH BSO, small and large bowel resection with end colostomy	Squamous cell carcinoma of the ovary	Nil	Palliative surgery for symptomatic relief
					Stage IV		1L EBL with 4 units RBC and 2 units FFP given intra-op
*4*	51	32	2	TLH BSO	Endometrial hyperplasia	Trachelectomy for cervical cancer	Significant adhesions mostly connecting omentum and large bowel to anterior abdominal wall
						Caesarean section	
						Hypertension	
*5*	51	46	3	TLH BSO and sentinel lymph node biopsy	Endometrial cancer	Type 2 diabetes	Nil
					Stage 1A	Atrial fibrillation taking apixaban	
						Hypertension	
						Gout	
*6*	49	38	3	TAH BSO	Risk reducing procedure for BRCA1	Type 2 diabetes	Laparoscopic procedure converted to open due to multifibroid uterus
						Obstructive sleep apnoea requiring CPAP	
						Hypertension	
						Previous uterine artery embolization	
*7*	54	30	2	TAH BSO	Unscheduled bleeding on HRT	Large multifibroid uterus	Rectum firmly adherent to posterior uterus
						2 previous Caesarean Sections	Bladder high on anterior uterus covering large fibroids
						Endometriosis	900mls EBL with 2 units RBC and FFP given intra-op
						Hypothyroidism	
*8*	79	42	3	TLH BSO and sentinel lymph node biopsy	Endometrial cancer	Heart failure	Nil
					Stage 1A	1st degree heart block	
						CKD	
*9*	55	61	3	TLH BSO	Atypical endometrial hyperplasia	Type 2 diabetes	Challenging anaesthetic ventilation requiring low pressure intra-operative pneumoperitroneum and minimal head-down positioning
						Obstructive sleep apnoea requiring CPAP	
						Splenic vein thrombosis	
						Recurrent cellulitis	

The diagnosis of an infected vaginal vault haematoma was made if patients displayed a vaginal vault haematoma of at least 2 cm on dedicated pelvic imaging combined with objective findings of infection. In addition to the vault haematoma, one of fever, raised inflammatory markers not in keeping with the recent surgery, purulent discharge or worsening pain must have been present. All patients underwent transvaginal ultrasound with first impression (pattern recognition) used to identify vault haematomas. No specific biochemical thresholds were set, however all patients diagnosed with an infected vaginal vault haematoma displayed rising inflammatory markers prior to drainage.

**Figure 1:**
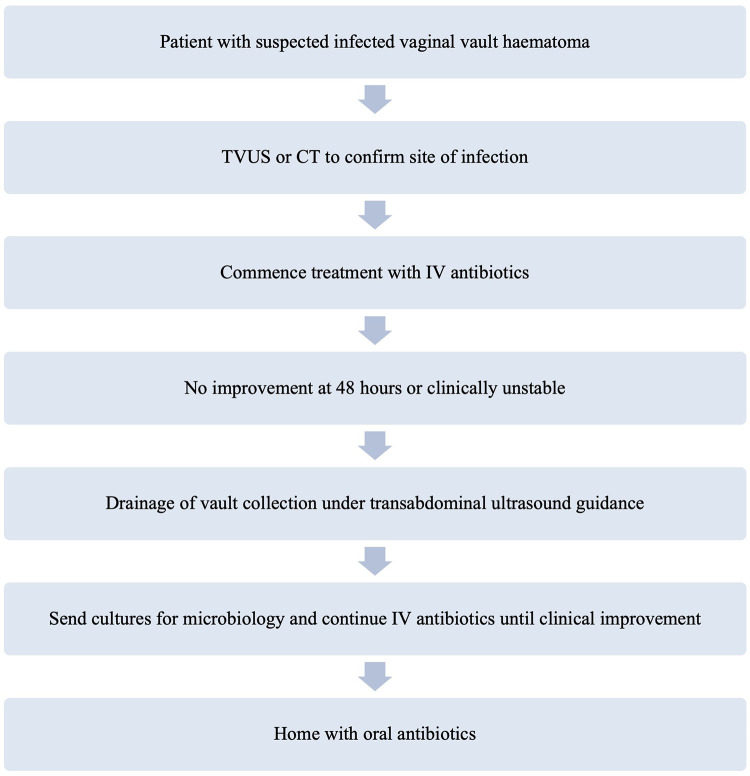
Flow diagram of proposed management pathway for patients presenting with an infected vaginal vault haematoma.

The patients were selected for drainage because they either did not respond adequately to intravenous broad-spectrum antibiotics (*n* = 8) or were only suitable for physical intervention (*n* = 1). The latter applied to patient 8, who had a non-infectious vault lymphocyst. Patients were deemed to have had an insufficient response to antibiotics if the previously described diagnostic criteria were not improving 48 h after commencement or if their clinical condition deteriorated prior to this. Patient 2 opted for initial outpatient drainage after initially presenting with an infected vault haematoma so did not have IV antibiotics prior this, however she represented and was admitted with intravenous antibiotics administered prior to the second drainage. Figure 1 displays a proposed pathway for the management of patients with suspected infected vault haematomas.

Patients were treated over a 24-month period spanning 2023–2025 during which 1,017 hysterectomies were performed, and 20 symptomatic vaginal vault collections were treated. Patients were treated with our proposed method (*n* = 9) or were treated conservatively with intravenous antibiotics. None had a return to theatre or interventional radiology drainage for an infected vaginal vault haematoma.

Our technique uses transabdominal ultrasound to guide the partial separation of the vaginal vault sutures. This is performed after the patient has undergone a detailed transvaginal ultrasound to elucidate the exact anatomical location of the collection, which must be communicating with the vaginal vault. An expert ultrasound must be performed prior to attempting drainage to ensure the correct diagnosis is reached. CT imaging may aid in the diagnostic process by investigating for other intra-abdominal pathology but is not essential prior to drainage.

**Figure 2:**
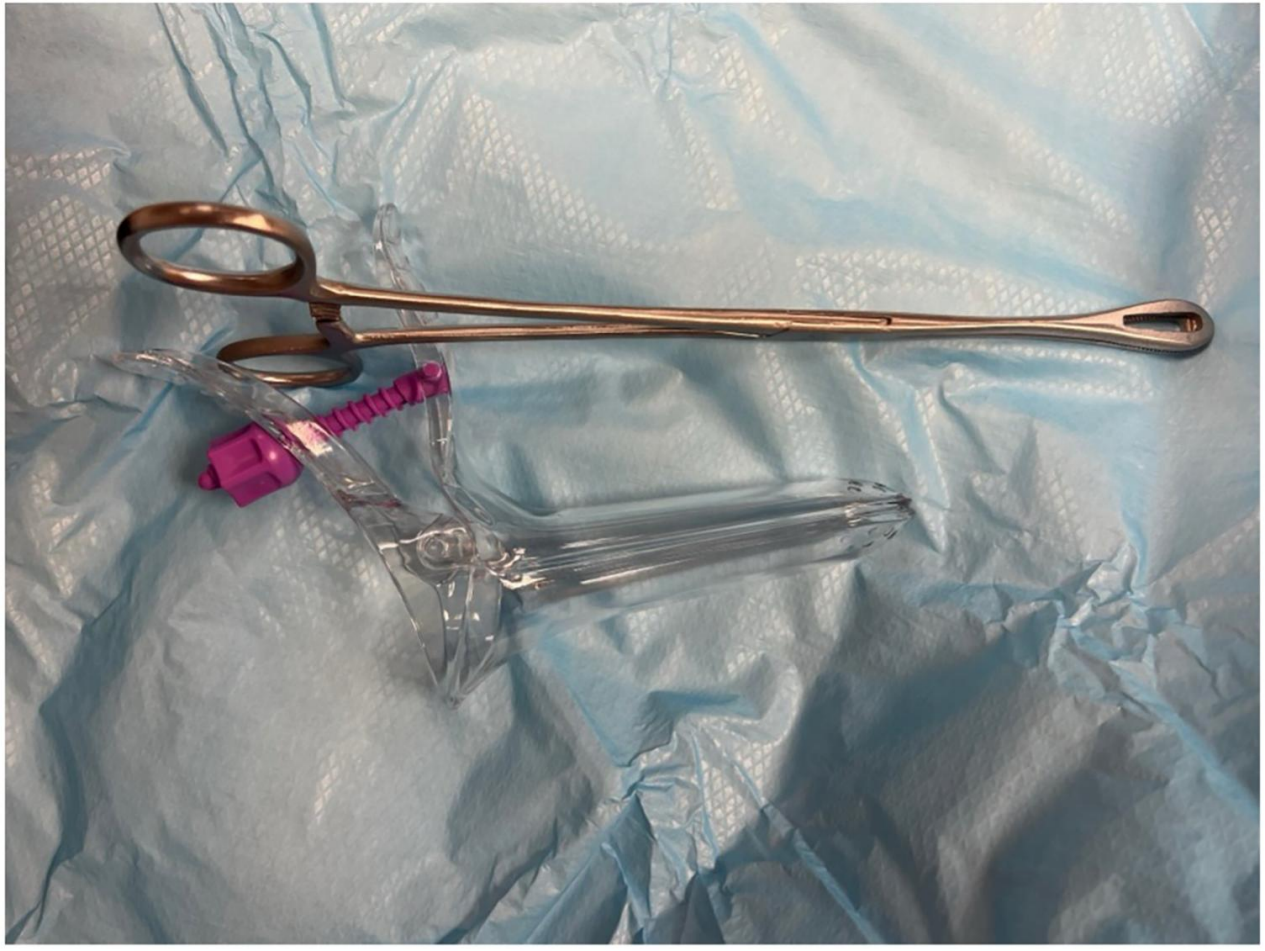
Equipment required for the described technique: **(i)** long, wide-jawed sponge holding forceps and **(ii)** disposable Cusco speculum.

**Figure 3:**
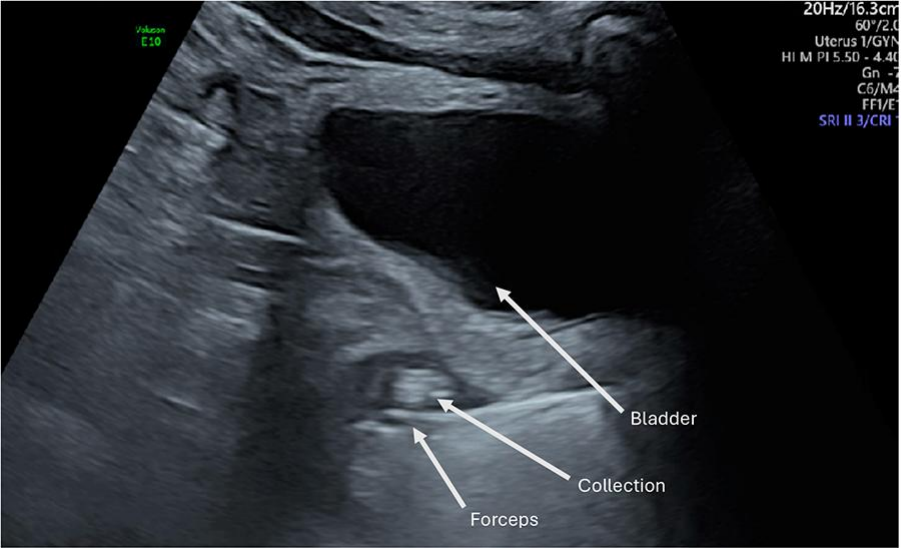
Transabdominal ultrasound image displaying forceps, infected vault collection and bladder. Sagittal view.

Once the diagnosis is confirmed, the patient is consented and placed into lithotomy position. A trained expert clinician will perform a transabdominal ultrasound to allow operator visualisation of the haematoma and vault. When the patient is comfortable, Cusco speculum examination is performed and local anaesthetic gel instilled into the vagina. A pair of wide-jawed polyp or sponge holding forceps is then gently introduced with the broad surface of the jaws positioned antero-posteriorly. Once the forceps are advanced through the vault, the jaws are gently opened, which in turn separates the sutures, allowing an opening of the vaginal vault, and the collected fluid to drain out per vagina. The use of ultrasound-guidance directs the forceps to the vault collection allowing complete drainage. The required equipment and typical transabdominal ultrasound findings are displayed in Figures 2, 3, respectively.

The presence of infection at the vaginal vault means the tissue can open with little force from the forceps and the patient remains relatively comfortable throughout. Maximum amount of fluid is allowed to drain, and gravity assists in completing the process of fluid drainage. No drains are required. This procedure takes on average 3–5 min and is well tolerated by patients with minimal discomfort. The Supplementary Videos demonstrate the transvaginal ultrasound findings of a vaginal vault collection (supplementary video S1) and a transabdominal ultrasound recording of the technique we describe (supplementary video S2).

In this article, “days” refer to the number of days post-surgery. For example, day 1 is the day after a patient underwent their operation.

All patients provided informed consent for their treatment to be described in this case series. No additional ethical approval was required for this case series, as confirmed by institutional policy.

## 3 Results

**Figure 4:**
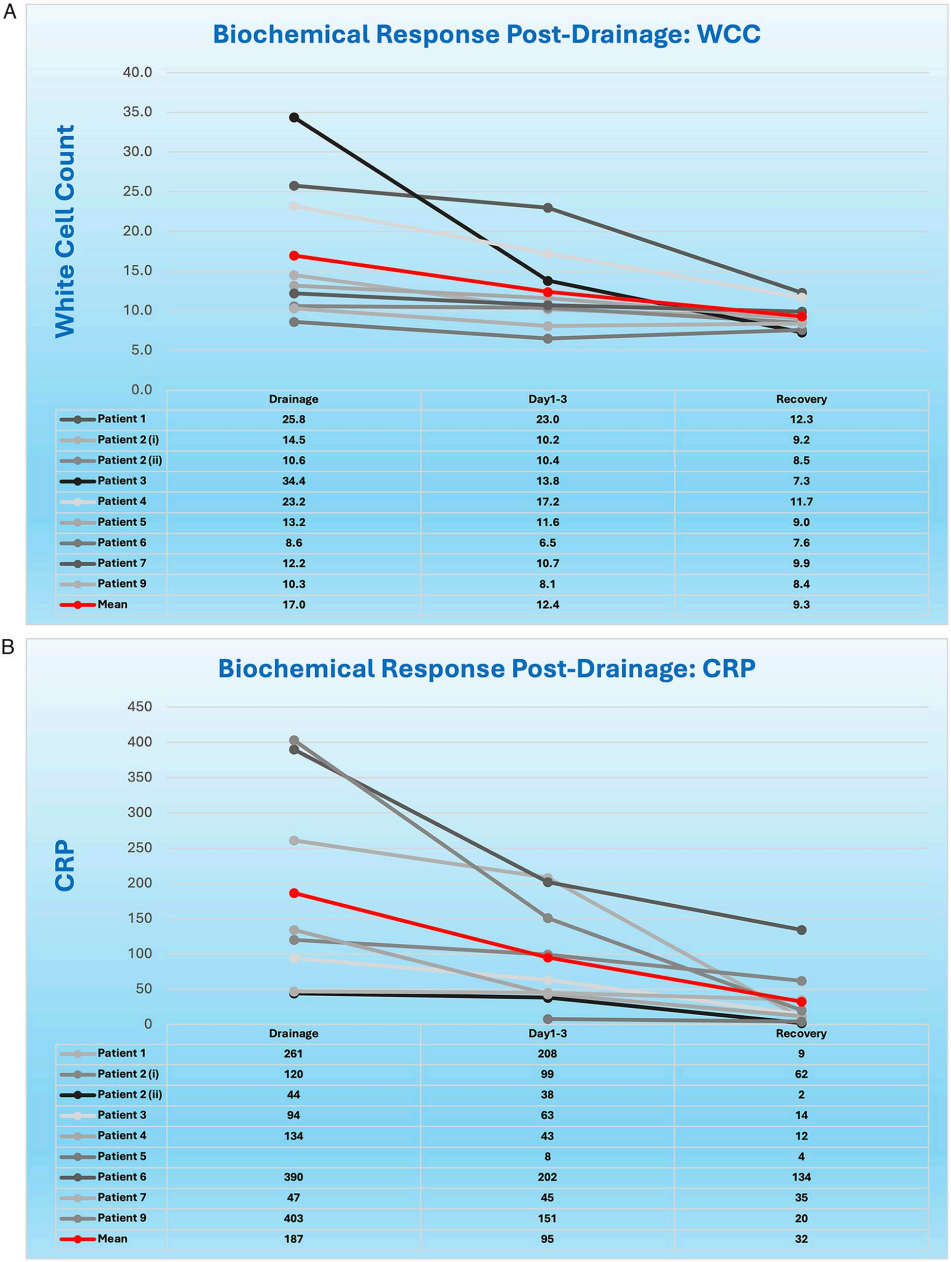
**(A)** Patients' WCC trend starting at drainage. Patient 2 underwent two drainages. Patient 8 had no bloods due to non-infectious lymphocyst. **(B)** Patients' CRP trend starting at drainage. Patient 2 underwent two drainages. Patient 5 did not have a CRP performed on the day of her drainage. Patient 8 had no bloods due to non-infectious lymphocyst.

The results for this case series are supplemented by tables (Tables 1–3, Supplementary Table 1) and figures (Figures 4A,B) that describe the intra- and post-operative course of nine patients who developed vaginal vault collections. Eight patients had an infected vault haematoma refractory to intravenous antibiotics and one patient developed a symptomatic vault lymphocyst. All patients had their collections successfully treated using the operative technique we describe. One patient (patient 2) required two drainages, both using the same technique.

**Table 2:** Patient post-operative course, imaging and culture results.

Patient	Post-op Course	Imaging	Culture	Images	
*1*	**Day 2** develops recurrent pyrexia	**Day 4 CTAP**	Drained fluid *E. Coli*	**Pre-drainage vault collection**	**Post-drainage vaginal vault**
	**Day 5** drainage (150mls malodorous fluid)	Infected collection anterior to rectum		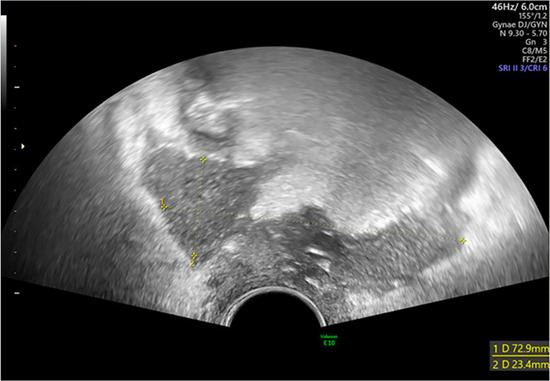	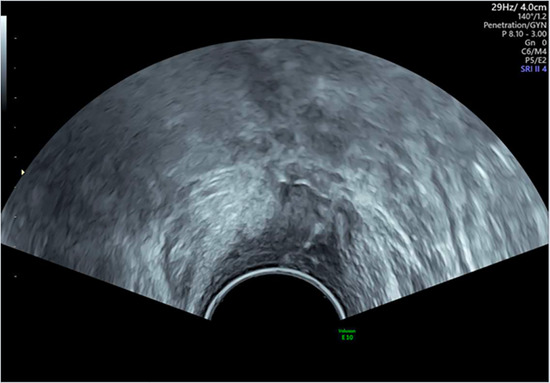
	**Day 6** pyrexia resolves	**Day 5 TVUS**			
	**Day 13** discharged	77 × 73 × 23 mm collection confirmed to communicate with vaginal vault			
*2*	**Day 3** hospital acquired pneumonia	**Day 15 TVUS**	Day 15	**Pre-drainage vault collection (repeat)**	**Post-drainage vaginal vault (repeat)**
	**Day 7** discharged	41 × 33 × 28 mm infected vault haematoma	Drained fluid	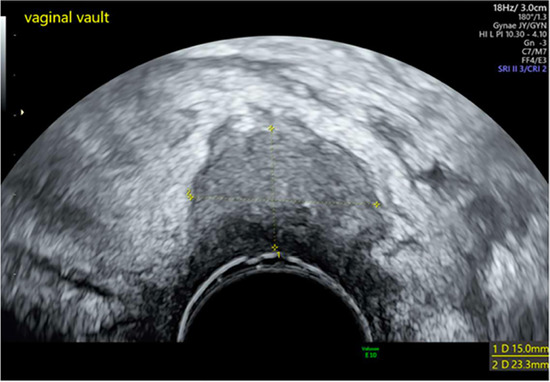	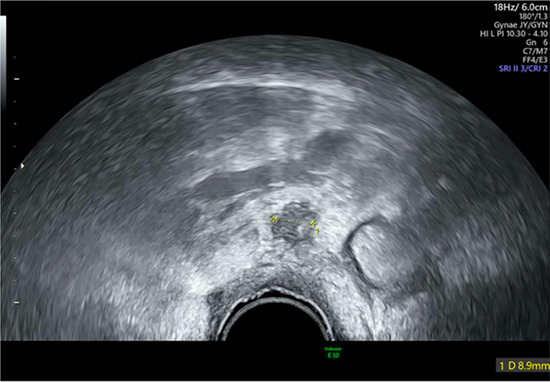
	**Day 15** pain and vomiting with initial vault drainage performed (outpatient)	**Day 23 CTAP**	*E. Coli*		
	**Day 23** readmission with pyrexia with recurrent vault haematoma	Collection within rectovesical pouch extending cranially from vaginal vault	Day 23		
	**Day 24** repeat drainage performed	**Day 24 TVUS**	N.R.		
	**Day 26** discharged	23 × 18 × 15 mm infected vault haematoma			
*3*	**Day 1** 150mls serous fluid in drain	**Day 7 TVUS**	Drained fluid	**Pre-Drainage**	**Post-Drainage**
	**Day 3** drain removed	80 × 80 mm pelvic collection connecting with vaginal vault	*Pseudomonas aeruginosa*	No images	No images
	**Day 7** significant WCC rise		Wound swab		
	**Day 7** haematoma drainage		*Pseudomonas aeruginosa*		
	**Day 11** discharged				
*4*	**Day 1** discharged (patient request)	**Day 8 CTAP**	Day 7 HVS	**Pre-drainage vault collection (CT Scan, sagittal)**	**Post-drainage vaginal vault**
	**Day 7** readmitted with pain and offensive vaginal discharge	Possibly connected collections: (i) right iliac fossa and (ii) at vaginal vault	*E. coli*	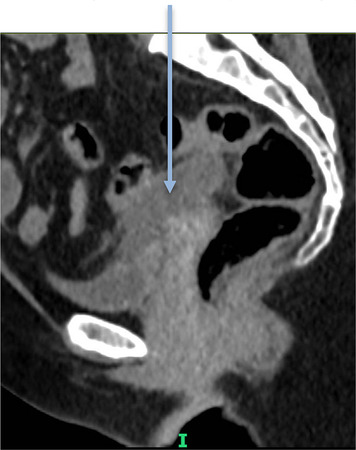	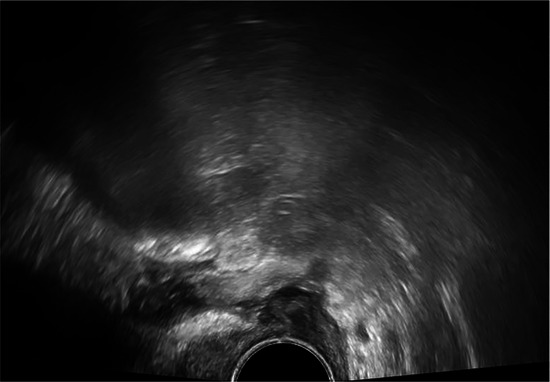
	**Day 9** drainage		*Anaerobes*		
	**Day 12** discharged		Drained fluid		
			*E. coli Enterococcus faecalis*		
*5*	**Day 2** discharged	**Day 13 TVUS**	Drained fluid	**Pre-drainage vault collection**	**Post-drainage vaginal vault**
	**Day 13** readmitted with offensive vaginal discharge and dysuria	73 × 36 × 32 mm infected vault haematoma	*E. coli* (ESBL)	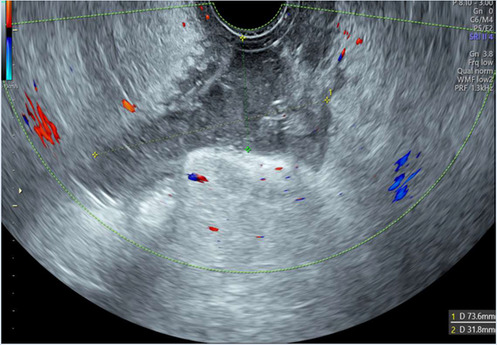	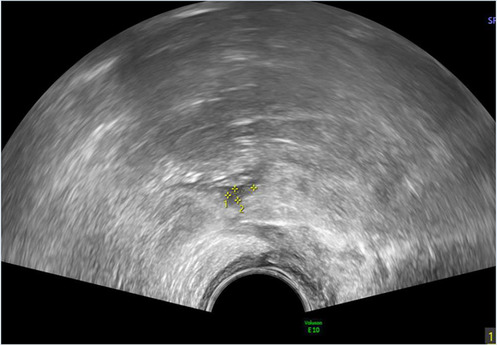
	**Day 15** drainage		*Morganella morganii (ESBL)*		
	**Day 17** discharged		*GBS*		
			*Anaerobes*		
*6*	**Day 2** developed pyrexia	**Day 4 TVUS**	Drained fluid	**Pre-drainage vault collection**	**Post-drainage vaginal vault**
	**Day 4** persistent pyrexia and very high CRP	74 × 63 × 33 mm infected vault haematoma	*Enterococcus faecalis*	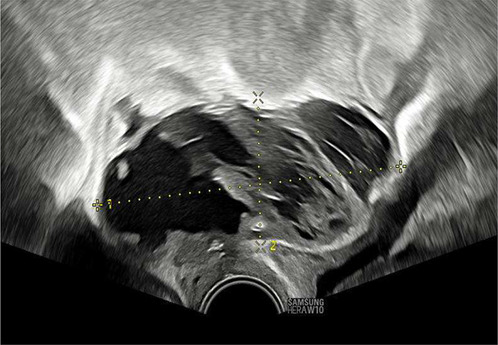	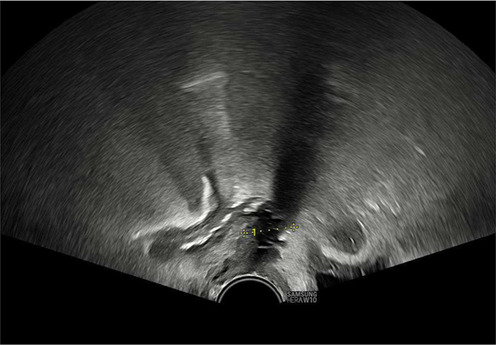
	**Day 4** drainage (200mls turbid fluid discharged)				
	**Day 7** discharged				
*7*	**Day 3** discharged	**Day 11 CTAP**	Drained fluid	**Pre-drainage vault collection (CT Scan, sagittal)**	**Pre-drainage vault collection (CT Scan, coronal)**
	**Day 11** readmission reporting 3 days of fevers and serosanginous vaginal discharge	77 × 65 mm vaginal vault collection	Negative microbiology	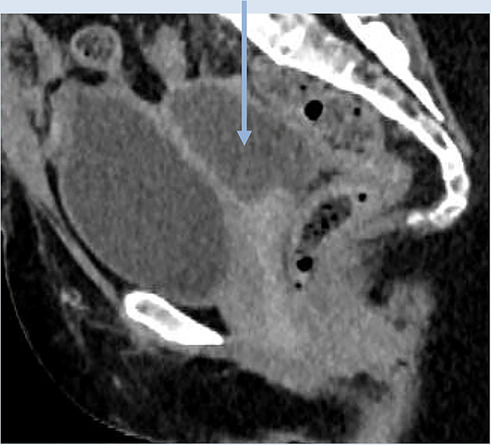	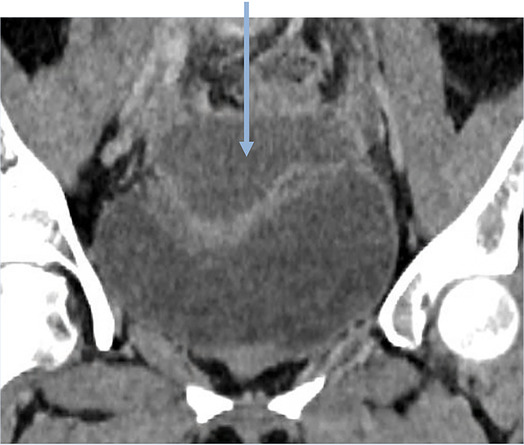
	**Day 14** drainage		Rectal swab		
	**Day 14** discharged		*E. Coli*		
			*(CPE NDM)*		
*8*	**Day 2** discharged	**Day 12 TVUS**	Drained fluid	**Pre-drainage vault lymphocyst**	**Pre-drainage vault lymphocyst 3D rendering**
	**Day 12** feels well but reports large volume non-odorous vaginal discharge	Likely lymphocyst	Negative microbiology	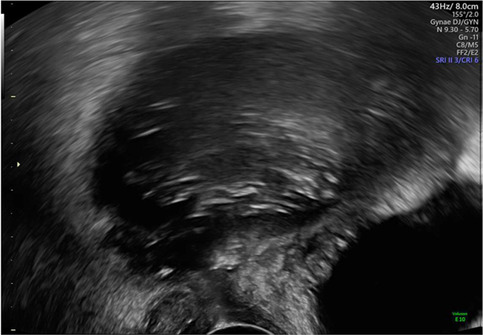	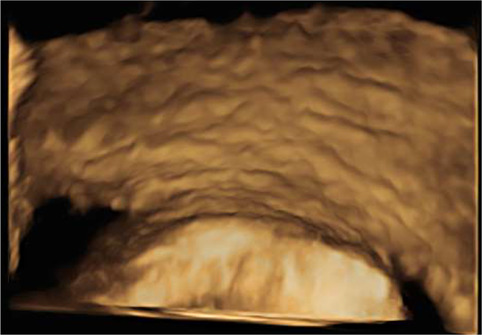
	**Day 12** vaginal vault intact with pooling clear fluid seen		Fluid creatinine 73 mol/L		
	**Day 12** drainage (outpatient)		(in keeping with lymph fluid)		
*9*	**Day 3** discharged	**Day 5 CTAP**	Drained fluid	**Pre-drainage vault collection**	**Post-drainage vaginal vault**
	**Day 5** readmission with abdominal pain and fevers	105 × 90 × 50 mm vaginal vault collection	*E. coli*	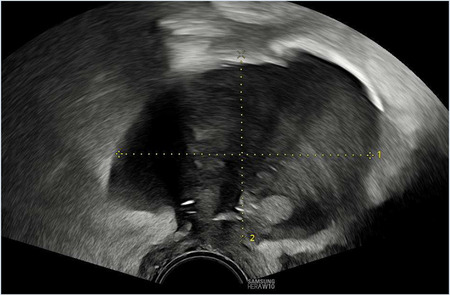	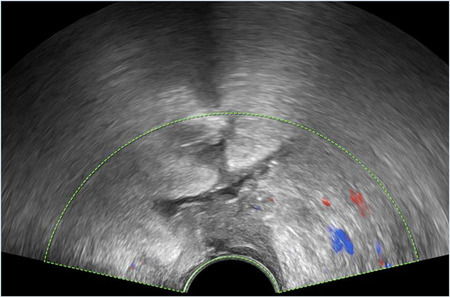
	**Day 7** malodorous vaginal discharge and refractory fevers	**Day 8 TVUS**	*Enterococcus faecalis*		
	**Day 8** drainage	65 × 48 × 70 mm	*Lactobacillus*		
	**Day 13** discharged	infected vault haematoma	*Corynebacterium*		
			*Staphylococcus epidermis*		

**Table 3:** Length of admission, days to symptom resolution, and follow-up duration.

Patient	Admission days	Days pre-drainage	Days post-drainage	Days to resolution of symptoms	Follow-up (months)
**1**	13	5	8	7	20
**2 (i)**	0 (outpatient)	0	0	5	15
**2 (ii)**	4	2	2	2	15
**3**	11	7	4	3	6
**4**	5	3	2	2	12
**5**	5	3	2	2	24
**6**	7	4	3	3	6
**7**	4	4	0	3	6
**8**	0	0	0	3	23
**9**	9	4	5	5	6

Admission days for patients 1, 3, 6 represent their entire hospital inpatient stay. If patients were re-admitted [patients 2 (ii), 4, 5, 7, 9] then only these days are reported and not days from the initial post-operative course. Patients 2 (i) and 8 had drainage performed in an outpatient setting so were not admitted. Days pre-drainage are inclusive of the day of drainage. Days post-drainage are from the day after drainage to discharge. Days to resolution of symptoms are measured from drainage to complete resolution of clinical features (e.g., pyrexia, pain, discharge), which may have exceeded the admission. Follow-up duration is the total outpatient monitoring period post-discharge. The median follow-up duration is 12 months.

Table 1 describes the patients' co-morbidities and primary surgery. All patients had more than one of a raised body mass index (BMI), ASA ≥2, significant surgical or medical history, established cancer or an intra-operative complication. These factors likely contributed to the formation of infected vaginal vault haematomas refractory to intravenous antibiotics.

As seen in Table 2, following surgery patients developed typical symptoms of infected vaginal vault haematomas including pain, pyrexia, fever and abnormal discharge. The patient with a lymphocyst remained clinically well but reported large volume non-odorous vaginal discharge. The collections requiring drainage, identified on imaging, varied in size from 23 mm to 80 mm in their largest dimension. All patients underwent transvaginal ultrasound prior to drainage with five patients also undergoing CT imaging as part of investigation for their acute illness.

Where available, imaging of the vaginal vault collections is demonstrated in Table 2 with a range of typical ultrasound and CT findings shown. Five patients have pre- and post-drainage imaging, showing the clear and immediate difference in appearance of the vaginal vault if drainage is successful and complete. Table 3 describes the length of admission for the symptomatic vault collections, time to symptom resolution and follow-up duration.

Seven of the eight patients with an infected haematoma had a positive culture result. Escherichia coli and Enterococcus faecalis were the most common organisms, found in five and three patients respectively. Three patients were found to have more than one pathogen. Patient 7 had a negative culture result from the drained vault fluid, however had a typical presentation of an infected vault haematoma and had a good clinical improvement following drainage.

Patients' biochemical response to drainage is outlined in Figures 4A,B, with a clear reduction in inflammatory markers following drainage demonstrated. Bloods were taken on the day of drainage, 24–72 h later and at the point of recovery, defined as the resolution of symptoms which occurred no later than seven days following drainage. Mean white cell counts (WCC) were 17.0, 12.4 and 9.3, and mean CRP levels were 187, 95 and 32 at these time points.

No procedure-related complications occurred. Complications were predefined as any of the following: intra-procedural bleeding requiring intervention or transfusion, visceral injury (bladder, bowel or ureter), worsening infection, unplanned return to theatre, or hospital readmission within 30 days attributable to the drainage procedure. All patients were followed-up in the gynaecology outpatient clinic until complete symptom resolution and radiological confirmation of vault collection resolution. All patients were followed-up for at least 6 months. No patients required additional interventions beyond the single repeat drainage in Patient 2.

## 4 Discussion

Here we describe a novel technique in a proof-of-concept case series that may aid patients and clinicians alike in the drainage of refractory vaginal vault collections. We have demonstrated a good radiological, clinical and biochemical response to treatment within the limitations of a small patient cohort. Our described technique adds to the range of ultrasound-guided gynaecological procedures already described in the literature, solidifying the essential role of ultrasound in improving procedural accuracy and reducing patient morbidity (21, 23).

Given most infected haematomas are found at the vaginal vault they are likely to be amenable to our proposed technique (7, 32). Unwell and high-risk patients, such as those with a very high BMI or a complex surgical history, are effectively treated and tolerate the procedure well in our experience. These patients may often have limited treatment options due to their co-morbidities and this offers a potential alternative to high-risk interventional radiology or surgical procedures. Patient 9 had initially represented to a different hospital after becoming unwell following surgery. This hospital had deemed her too high risk for an interventional radiology procedure given her BMI of 61 and clinical condition. As such, a return to theatre was being considered. We arranged transfer back to our hospital where we successfully drained vault collection in a ward setting, avoiding a high-risk surgical procedure.

Case reports from the 1980s and 1990s describe transvaginal techniques to drain vault haematomas under transabdominal ultrasound-guidance as alternatives to surgical interventions including colpotomy, laparotomy, pelvic washout and transabdominal drainage (33, 34). An example is the insertion of a large bore cannula into well circumscribed haematomas, draining the infected fluid and then performing pelvic irrigation (33). An alternative approach described inserting a digit through the vaginal vault into an infected haematoma to manually break down locules, followed by insertion of a Foley catheter into the collapsed space to allow further drainage (34). Finally, transrectal ultrasound was proposed to guide the opening of vaginal vault sutures for the subsequent drainage of an infected vault haematoma (35).

Return to theatre for exploratory procedures requiring an anaesthetic has significant additional risks. Treatment of patients with multiple co-morbidities, challenging primary operations and those that have undergone complex cytoreductive procedures requires careful planning. These patients are often already in significant distress making pain reduction an integral part of their management. In this context, a comparison can be made to digital opening of the vaginal vault, with or without ultrasound-guidance, to facilitate drainage. However, manual examination of the vagina is more invasive and uncomfortable.

A report on nine out of 648 post-hysterectomy patients who required drainage of an infected vault haematoma describes seven that had this satisfactorily performed by the introduction of a cotton swab through the vaginal vault (7). This technique is also likely to minimise discomfort when compared with digitally opening the vault. Ultrasound-guidance would likely add further benefit due to the precision it affords.

Segev et al. describe nine out of 308 women who following vaginal hysterectomy underwent drainage of an infected haematoma under general anaesthesia (32). They used transvaginal ultrasound-guidance for the opening of vaginal vault sutures and the placement of a suction device into the haematoma to evacuate it. Whilst effective, this carries both the risks of a general anaesthetic and surgical suction devices.

Another proposed technique used in 16 patients with post-hysterectomy pelvic collections is a trocar or Seldinger method to puncture the vagina and introduce a pigtail catheter into the collection (36). While the authors report a 100% success rate this method requires additional equipment, conscious sedation for some patients and bladder filling, sometimes with re-catheterisation, and a high level of expertise. The authors report no major complications, however this technique may lend itself to a greater range of adverse events given the equipment and medications required. An advantage of this method is that it allows treatment of collections that do not connect with the vaginal vault.

Infected collections communicating with a vaginal incision may also be seen following the extraction of surgical specimens via colpotomy and from the minimally invasive vaginal natural orifice transluminal endoscopic surgery (VNOTES) approach to gynaecological and general surgical procedures (37). While infected collections in this setting may arise from a different disease process, minimally invasive ultrasound-guided approaches to drainage similar to what we describe may be adopted as these procedures increase in popularity.

Finally, we found Escherichia coli and Enterococcus faecalis to be the most common organisms in haematoma culture, and we found three patients had more than one pathogen isolated, all of which are consistent with the literature (7).

### 4.1 Limitations

This proof-of-concept case series has several limitations given its structure and cohort, as we report on a small patient number with no comparator group. This means the effectiveness of our proposed technique over current methods, such as those described above, or conservative management cannot be ascertained. Similarly, while we report no adverse events, the safety and complication rate of this technique cannot be accurately reported on with our small patient number. Comparison to other techniques described in the literature is limited by both our small sample size and the small sample size of the other approaches described.

Further to these limitations it should be noted that all patients had drainage performed by a highly skilled operator trained in advanced ultrasound and gynaecological surgery, working in a tertiary gynaecological oncology referral unit. As such, it is not possible to extrapolate the safety or effectiveness of performing this technique by less skilled operators and in a less specialised or lower resource setting. While we have found that this procedure is easily understood and adopted by junior staff, we acknowledge this is under expert supervision and therefore may not be replicable in other settings. Finally, not all these patients have undergone long term follow-up to assess for procedure-specific late complications (range 6–24 months), however none represented with symptoms of infected vault haematoma or other complications following their treatment course described here.

## 5 Conclusions

We have presented a simple, low-cost, non-surgical method of draining pelvic collections that communicate with the vaginal vault post-hysterectomy. This approach may be particularly beneficial for cases where infection is present as it avoids major intervention in the already unwell and often co-morbid patient. We have observed this to be well tolerated by patients, anecdotally causing less discomfort than other techniques previously adopted in our unit. Broader adoption may be considered by adequately trained gynaecologists familiar with ultrasound-guided procedures subject to further studies confirming efficacy and safety.

## Data Availability

The raw data supporting the conclusions of this article will be made available by the authors, without undue reservation as long as patient confidentiality is not at risk.

## Supplementary material

Supplementary Video S1 Ultrasound recording of a post-operative collection connecting with the vaginal vault.

Supplementary Video S2 The described technique being performed with sponge holding forceps under transabdominal ultrasound-guidance. The opening and closing of the forceps within the collection to allow full drainage is safely performed under ultrasound-guidance.
