# Supplementary material for: An ultrasound-guided minimally invasive treatment for vaginal vault collections following major gynaecological surgery: a case series
Source: Front Surg. 2026 May 11;13:1661216. doi: 10.3389/fsurg.2026.1661216 (PMC13199716; doi:10.3389/fsurg.2026.1661216)
Supplement: Supplementary file 1 [file Table1.docx]

**Key for Tables and Figures:**

TLH BSO – Total laparoscopic hysterectomy and bilateral salpingo-oophorectomy

PMDD – Premenstrual dysphoric disorder

VTE – Venous thromboembolism

TAH BSO – Total abdominal hysterectomy and bilateral salpingo-oophorectomy

EBL – Estimated blood loss

RBC – Red blood cells

FFP – Fresh frozen plasma

BRCA1 – BReast CAncer gene 1

CPAP – Continuous positive airway pressure

HRT – Hormone replacement therapy

CKD – Chronic kidney disease

CTAP – CT Abdomen and Pelvis

TVUS – Transvaginal ultrasound scan

*E. coli*  – Escherichia coli

N.R. – Not recorded

WCC – White cell count

HVS – High vaginal swab

ESBL – Extended spectrum Beta-Lactamase

GBS – Group B Streptococcus

CRP – C reactive protein

CPE – Carbapenemase-producing Enterobacteriaceae

NDM – New Delhi metallo-beta-lactamase 1
